# Association between oxidative balance score and diabetic kidney disease, low estimated glomerular filtration rate and albuminuria in type 2 diabetes mellitus patients: a cross-sectional study

**DOI:** 10.3389/fendo.2024.1412823

**Published:** 2024-07-31

**Authors:** Cong Liu, Jiju Yang, Hongdian Li, Yuanyuan Deng, Pengfei He, Jiao Zhang, Mianzhi Zhang

**Affiliations:** ^1^ Department of Nephrology, Dongfang Hospital of Beijing University of Chinese Medicine, Beijing, China; ^2^ Graduate School of Beijing University of Chinese Medicine, Beijing, China; ^3^ Department of Nephrology, Tianjin Academy of Traditional Chinese Medicine Affiliated Hospital, Tianjin, China; ^4^ Tianjin Famous Chinese Medicine Inheritance Workshop of Mianzhi Zhang, Tianjin, China

**Keywords:** diabetic kidney disease, oxidative balance score, type 2 diabetes mellitus, NHANES, cross-sectional study

## Abstract

**Objective:**

The oxidative balance score (OBS) is a comprehensive concept that includes 20 oxidative stressors and can be used to assess individual pro-oxidant versus antioxidant exposure, and the aim of the present study was to investigate the association between OBS and the risk of diabetic kidney disease (DKD), low estimated glomerular filtration rate (low-eGFR) and albuminuria in patients with diabetes mellitus (DM).

**Methods:**

This cross-sectional study included nationally representative consecutive National Health and Nutrition Examination Survey DM patients aged 18 years and older from 2003-2018. The continuous variable OBS was converted into categorical variables by quartiles, and weighted multiple logistic regression analyses and restricted triple spline models were used to explore the relationships. We also performed subgroup analyses and interaction tests to verify the stability of the results.

**Results:**

A total of 5389 participants were included, representing 23.6 million non-institutionalized US residents. The results from both multivariate logistic regression analysis and restricted cubic spline models indicated that OBS and dietary OBS levels were negatively associated with the risk of DKD, low-eGFR, and albuminuria, without finding a significant correlation between lifestyle OBS and these clinical outcomes. Compared to the lowest OBS quartile group, the prevalence risk of DKD (OR = 0.61, 95% CI: 0.46-0.80), low-eGFR (OR = 0.46, 95% CI: 0.33-0.64) and albuminuria (OR = 0.68, 95% CI: 0.51-0.92) decreased by 39%, 54% and 32%, respectively, in the highest OBS quartile group. The results remained stable in subgroup analyses and no interaction between subgroups was found.

**Conclusion:**

Higher levels of OBS and dietary OBS were associated with a lower risk of DKD, low-eGFR, and albuminuria. These findings provided preliminary evidence for the importance of adhering to an antioxidant-rich diet and lifestyle among individuals with diabetes.

## 1 Introduction

Diabetes Mellitus (DM) is a major disease that endangers human health. According to the International Diabetes Federation, it is estimated that the number of people affected by DM will reach 1.09 billion by 2045 (1), leading to a corresponding increase in the prevalence of diabetic kidney disease (DKD). DKD is responsible for 30% to 50% of end-stage renal disease cases worldwide (2). The burden of DKD is significant, leading to reduced quality of life, increased incapacity, premature death (3), and higher healthcare costs (4). Therefore, it is still imperative to address the urgent issues of early prevention and slowing down the progression of DKD, as well as gaining a comprehensive understanding and effectively controlling the risk factors for its development.

The pathogenesis of DKD is complex (5), and oxidative stress is one of the important triggers (6). Oxidative stress is characterized by an overproduction of reactive oxygen species (ROS) that surpasses the body’s antioxidant defense system, resulting in an imbalance in the oxidative system. Hyperglycemia can result in the formation of advanced glycation end-products and ROS. These, in turn, activate intercellular signaling pathways that lead to pro-inflammatory and pro-fibrotic gene expression, causing tissue and cellular damage (7, 8). It is critical to maintain the balance of the body’s redox system to slow the progression of DKD (9). These findings have also sparked exploration into whether the intake of antioxidants can reduce the risk of DKD. However, the results are not entirely consistent (10–12), which is associated with the presence of various antioxidant and pro-oxidant factors in diet and lifestyle. Conclusions drawn from a sole evaluation of a specific factor may be one-sided.

The oxidative balance score (OBS) is a comprehensive indicator that considers various dietary components and lifestyle factors to assess an individual’s exposure to pro-oxidants and antioxidants. A higher OBS indicates a stronger antioxidant capacity. Since oxidative stress is an important pathogenic mechanism in DKD, managing and improving oxidative balance may be a crucial strategy for the prevention and treatment of DKD. This includes consuming an adequate amount of antioxidants in the diet and maintaining a healthy lifestyle. Although the OBS has been widely used in numerous studies and a higher OBS has been associated with a lower prevalence and incidence of chronic kidney disease (CKD) (13, 14), as well as various diseases such as chronic obstructive pulmonary disease (15), kidney stones, cardiovascular disease (CVD) (16), and depression (17), research on the relationship between OBS and DKD is currently lacking. Given that individuals with diabetes are among the most susceptible populations to kidney damage, the presence of DKD complicates diabetes management, increases the risk of CVD, and mortality. The primary objective of this study is to utilize data from the National Health and Nutrition Examination Survey (NHANES) to conduct a cross-sectional analysis among the diabetic population to explore the association between OBS and DKD. The aim is to provide new insights and strategies for the prevention and treatment of DKD.

## 2 Materials and methods

### 2.1 Study population

This investigation is a nationwide cross-sectional study, utilizing data from the NHANES 2003-2018 survey cycle. NHANES is administered by the National Center for Health Statistics, a division of the Centers for Disease Control and Prevention, and has been approved by an ethics review board. The NHANES sample design incorporates a multi-year, stratified, clustered four-stage sampling approach, with data released in 2-year cycles (adjustments to the sampling methods for each cycle can be accessed via the following URL: https://wwwn.cdc.gov/nchs/nhanes/analyticguidelines.aspx#sample-design). Investigators gather comprehensive information from participants through home interviews and laboratory testing to assess the health and nutritional status of the non-institutionalized civilian population across the United States (18, 19), and all participants have provided informed consent.

**Figure 1:**
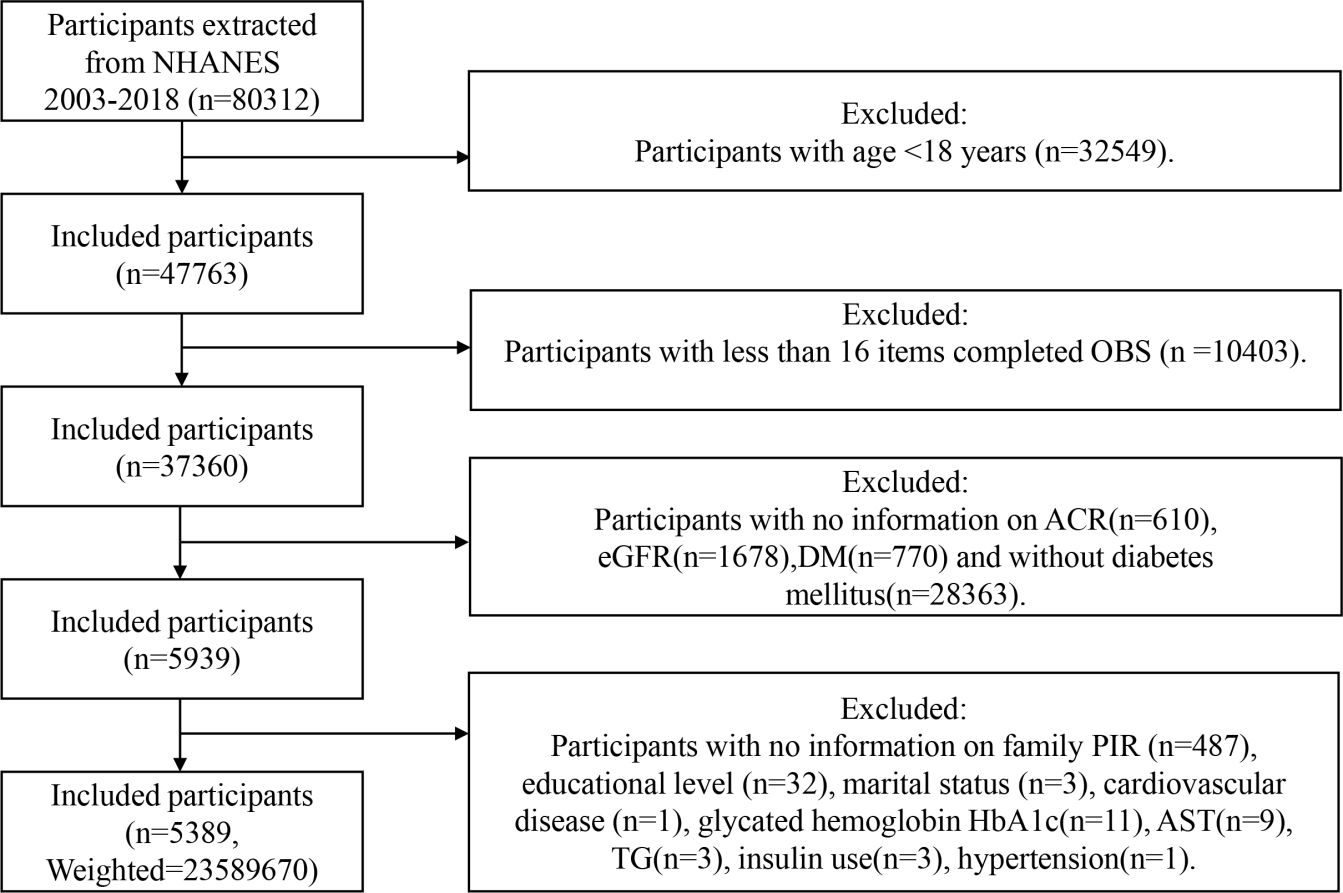
Flowchart of the sample selection from NHANES 2003–2018. NHANES, National Health and Nutrition Examination Survey; OBS, oxidative balance score; family PIR, family poverty income ratio; ACR, urinary albumin-to-creatinine ratio; DM, diabetes mellitus; eGFR, estimated glomerular filtration rate; AST, aspartate aminotransferase; TG, triglycerides.

Participants in this study underwent screening based on the following criteria (**Figure 1**): individuals were excluded if (1) <18 years; (2) Items comprising the OBS <16 (16); (3) incomplete data of urinary albumin-to-creatinine ratio (ACR); (4) incomplete data of estimated glomerular filtration rate (eGFR); (5) without DM; (6) pregnant; (7) incomplete data of covariates.

### 2.2 Definition of the oxidative balance score

The OBS is based on 16 dietary nutrients and 4 lifestyle factors. The 16 dietary nutrients comprise of dietary fiber, carotenoids, riboflavin, niacin, vitamin B6, total folate, vitamin B12, vitamin C, vitamin E, calcium, magnesium, zinc, copper, selenium, total fat, and iron. The 4 lifestyle factors include physical activity, body mass index (BMI), alcohol consumption, and smoking status. The study identified pro-oxidant factors, including total fat, total iron intake, smoking status, alcohol consumption status, and BMI. The remaining factors were classified as antioxidants (20). Food type and quantity intake were obtained by two 24-hour dietary recall interviews (the first assessment was administered at the Mobile Examination Center, followed by a telephone follow-up for the second assessment. The mean value of the data from both assessments was calculated, in the event of missing data from the second interview, the results from the first interview were considered as the default.), and based on the Food Intake Analysis System of the University of Texas and the United States Department of Agriculture Survey Nutritional Database. The amount of physical activity was calculated as the total metabolic equivalent of task (MET) level of all exercises performed in a week (metabolic equivalent per physical activity × frequency of physical activity × duration). The smoking level was determined by the cotinine level. Alcohol consumption was categorized into three groups based on gender: non-drinkers, moderate drinkers (0-15 g/d for women and 0-30 g/d for men), and heavy drinkers (≥15 g/d for women and ≥30 g/d for men). Obesity scores were assigned based on weight status, obesity: BMI≥30 kg per square meter (kg/m^2^), overweight: 25≤BMI<30 kg/m^2^, and normal weight: BMI<25 kg/m^2^. The OBS was calculated as follows (refer to **Supplementary Table 1**): non-drinkers, moderate drinkers, and heavy drinkers were scored 2, 1, and 0, respectively; obesity, overweight, and normal weight were scored 0, 1, and 2, respectively. The other components were grouped in tertiles by gender, with antioxidants in groups 1 - 3 being assigned a score of 0-2 and pro-oxidant factors in groups 1-3 being assigned a score of 2-0. Taking fiber intake as an example in men, daily fiber intake of ≤12.7g is assigned 0 point, between 12.7 and 19.85g is 1 point, and >19.85g is 2 points. Each component contributes an equal weight, and the sum of the scores yields the OBS. A higher OBS indicates a stronger antioxidant profile.

### 2.3 Definition of DKD, low-eGFR, and albuminuria

The diagnosis of diabetes (21): the use of diabetes medication or insulin, a diagnosis by a doctor, glycated hemoglobin HbA1c ≥6.5%, fasting glucose ≥7 mmol/L, 2-h oral glucose tolerance test with blood glucose ≥11.1 mmol/L. The DKD was defined as the diabetes combined with albuminuria (ACR≥30 mg/g) and/or low-eGFR (eGFR<60 mL/min/1.73 m^2^) according to the KDIGO 2021 Guidelines (22). For eGFR calculation, we applied the CKD-Epidemiology Collaboration equation as follows (23): GFR = 141·min [Scr/κ,1]α × max [Scr/κ, 1] − 1.209 × 0.993 Age × 1.018 [if women] ×1.159 [if black]); κ was 0.7(women) or 0.9(men), a was −0.329 (women) or −0.411(men), and min/max indicate the minimum/maximum of Scr/κ or 1.

### 2.4 Covariates assessment

Based on previous research (24, 25), we included a number of covariates that may influenced the results of the study. Demographic factors included age, gender, marital status (divorced/separated/widowed, married/living with a partner, never married), race/ethnicity (non-Hispanic white, non-Hispanic black, Mexican American and other), education level (less than high school, high school diploma, more than high school), the family poverty income ratio (PIR) (<1.3, 1.3-3.5, >3.5); laboratory indicators including aspartate aminotransferase (AST), alanine aminotransferase (ALT), total cholesterol (TC), triglycerides (TG), albumin (ALB), fasting glucose, glycated hemoglobin HbA1c; Additionally, we considered a number of chronic diseases: hypertension (taking blood pressure-lowering medication, diagnosed by a physician as having a high systolic blood pressure ≥140mmhg or diastolic blood pressure ≥90mmhg) (26), hyperlipidemia (taking cholesterol-lowering drugs, total cholesterol ≥200 mg/dL, triglyceride ≥150 mg/dL, low-density lipoprotein ≥130 mg/dL, or high-density lipoprotein ≤50 mg/dL for women and ≤40 mg/dL for men) (27), CVD (coronary heart disease, congestive heart failure, heart attack, stroke, angina) and metabolic syndrome(Mets), additionally insulin use.

### 2.5 Statistical analyses

In light of the stratified and complex multistage sampling design employed by NHANES, we have weighted the data using the sample weight calculation method recommended by NHANES. This involved pooling data from eight cycles spanning the period from 2003 to 2018, where the weights for 16 years are calculated to be one-eighth of the weights for 2 years. This weighting approach corrects for the imbalance in the samples, allowing for a more accurate reflection of the characteristics of the overall population. Categorical variables are presented as weighted percentages, while continuous variables are described using mean ± standard deviations. The Shapiro-Wilk statistical test was employed to confirm the normal distribution of continuous variables. Variables with skewed distributions are represented by medians and quartiles. We converted the continuous variable OBS into categorical variables by quartiles (Q1: ≤P25; Q2: P25-P50; Q3: P50-P75; Q4: >P75) and assessed differences between OBS (quantile) groups using weighted t-tests or chi-square tests. Multiple logistic regression is suitable for analyzing the relationship between multiple predictor variables and a binary outcome variable, allowing for the control of other potential confounding variables. Weighted multivariate logistic regression analysis was employed to evaluate the correlation between OBS, dietary OBS, lifestyle OBS, and DKD, low-eGFR, as well as albuminuria across three models. The results were reported as odds ratios (ORs) with their 95% confidence intervals (95% CIs), *p*-values, and trend *p*-values. Collinearity among variables was assessed using the variance inflation factor (VIF). The VIF quantifies the increased variance of an estimated regression coefficient due to collinearity. A common rule of thumb is that VIF values greater than 5 indicate a problematic level of collinearity (28), which can lead to instability in regression results and weaken predictive capabilities. In Model 1, adjustments were made for gender, age, marital status, race/ethnicity, education level, and family PIR. Model 2 adjusted for hypertension, hyperlipidemia, CVD, Mets, and insulin use based on Model 1. Model 3 adjusted for all covariates. A multivariate-adjusted restricted cubic spline model was employed to more accurately capture the relationship between continuous variables and the outcome. The model established OR curves at three knot points to explore whether there exists a nonlinear dose-response association between OBS and DKD, low-eGFR, and albuminuria. To determine the potential effect moderators, subgroup analyses were performed based on subjects’ age, sex, and whether they had hypertension, hyperlipidemia, CVD, and Mets, and interaction analyses were performed to check the heterogeneity of the relationship between subgroups. A two-sided *P* value of <0.05 was considered a statistically significant difference. R software (R version 4.3.3) was used for all statistical analyses in this study.

## 3 Results

### 3.1 Baseline characteristics

The study included 5389 participants, representing 23.6 million non-institutionalized residents of the United States. The mean age of all subjects was 59.57, with 50.37% males and 39.63% females. Among DM patients, the weighted prevalence of DKD, low-eGFR, and albuminuria was 36.57%, 18.38%, and 25.82%, respectively. Furthermore, we discovered significant differences (*p* < 0.05) in race, education level, marital status, family PIR, ALT, ALB, prevalence of hypertension, CVD and Mets, and insulin use among the OBS groups. **Table 1** presents the baseline characteristics of the study participants, categorized by their OBS quartiles.

**Table 1:** Characteristics of participants by quartiles of the OBS in the NHANES 2003–2018 cycles.

Variables	Total	Q1, [3, 13]	Q2, (13,18]	Q3, (18,24]	Q4, (24,35]	*p* value
	n = 23589670	n = 5424960	n = 5207344	n = 6636816	n = 6320550	
Age, years	59.57 ± 13.58	60.12 ± 13.52	60.32 ± 13.86	50.48 ± 13.92	58.56 ± 12.97	0.0626
Sex						0.5359
Male	50.37	52.57	49.19	50.92	48.89	
Female	49.63	47.43	50.81	49.08	51.11	
Race and ethnicity						< 0.0001
Non-Hispanic White	64.95	59.67	62.90	65.38	70.72	
Non-Hispanic Black	13.49	20.10	15.59	11.18	8.49	
Mexican American	8.79	8.05	9.57	9.27	8.28	
Other	12.77	12.18	11.94	14.16	12.50	
Educational level						< 0.0001
Less than high school	22.57	34.14	24.16	20.28	13.74	
High school diploma	26.20	27.79	28.99	23.25	25.62	
More than high school	51.23	38.06	46.85	56.46	60.64	
Marriage status						0.0144
Divorced/separated/widowed	27.60	31.10	28.84	28.29	22.84	
Married/living with a partner	64.02	59.04	62.82	63.59	69.73	
Never married	8.38	9.85	8.34	8.12	7.43	
Family PIR						< 0.0001
<1.3	24.85	36.39	28.07	21.15	16.19	
1.3-3.5	39.91	39.12	44.55	39.58	37.10	
≥3.5	35.24	24.49	27.38	39.27	46.71	
Hyperlipidemia						0.738
Yes	88.93	88.85	88.9	88.16	89.82	
No	11.07	11.15	11.10	11.84	10.18	
Hypertension						0.0107
Yes	70.75	73.73	74.72	68.47	67.31	
No	29.25	26.27	25.28	31.53	32.69	
Cardiovascular disease						< 0.0001
Yes	24.97	31.77	24.67	24.02	20.38	
No	75.03	68.23	75.33	75.98	79.62	
Metabolic syndrome						0.0184
Yes	76.86	76.30	79.37	79.14	72.86	
No	23.14	23.70	20.63	20.86	27.14	
Insulin use						0.0431
Yes	19.17	18.89	23.06	17.00	18.46	
No	80.83	81.11	76.94	83.00	81.54	
Glycohemoglobin, %	7.06 ± 1.60	7.11 ± 1.71	7.12 ± 1.58	7.01 ± 1.56	7.03 ± 1.56	0.3577
Fasting glucose, mmol/L	8.00 ± 3.56	8.02 ± 3.74	8.16 ± 3.60	7.95 ± 3.40	7.90 ± 3.51	0.6039
AST, U/L	23(19, 29)	23(19, 28)	23(19, 29)	23(19, 29)	23(19, 29)	0.2665
ALT, U/L	23(17, 31)	21(16, 30)	23(17, 32)	23(18, 31)	24(18, 31)	0.0005
TC, mmol/L	4.88 ± 1.24	4.95 ± 1.34	4.91 ± 1.24	4.87 ± 1.22	4.80 ± 1.19	0.2921
TG, mmol/L	1.81(1.22, 2.62)	1.74(1.23, 2.63)	1.83(1.22, 2.57)	1.91 (1.24, 2.69)	1.74(1.20, 2.62)	0.2162
ALB, g/dL	4.15 ± 0.33	4.09 ± 0.34	4.14 ± 0.33	4.18 ± 0.33	4.18 ± 0.32	0.0001
ACR, mg/g	11.30(6.28, 31.50)	13.74(7.09, 50.00)	11.39(6.55,30.40)	11.20(5.98, 26.88)	10.00(5.77,23.29)	<0.0001
Albuminuria						<0.0001
Yes	25.82	34.26	25.35	23.53	21.36	
No	74.18	65.74	74.65	76.47	78.64	
eGFR, mL/min/1.73 m^2^	82.93 ± 24.19	79.62 ± 26.55	81.11 ± 25.47	84.07 ± 23.11	86.06 ± 21.47	<0.0001
Low-eGFR						<0.0001
Yes	18.38	23.52	23.40	16.59	11.73	
No	81.62	76.48	76.60	83.41	88.27	
DKD						<0.0001
Yes	36.57	45.20	40.05	34.19	28.80	
No	63.43	54.80	59.95	65.81	71.20	

OBS, oxidative balance score; Q, quartiles; NHANES: National Health and Nutrition Examination Survey; DKD, diabetic kidney disease; Family PIR, family poverty income ratio; TC, total cholesterol; TG, triglycerides; ALT, alanine aminotransferase; AST, aspartate aminotransferase; ALB, albumin; ACR, urinary albumin-to-creatinine ratio; eGFR, estimated glomerular filtration rate.

Data presented as weighted percentage for categorical variables and mean+ SD for continuous variables, and skewed distribution variables as medians and quartiles.

### 3.2 Association between OBS, dietary OBS, lifestyle OBS with DKD, low-eGFR and albuminuria

As shown in **Supplementary Table 2**, all covariates exhibit VIF values less than 5, suggesting that collinearity has a minimal impact on the results.

As depicted in **Tables 2**–**4**, weighted multivariate logistic regression was employed to examine the association between OBS and the risk of DKD, low-eGFR, and albuminuria. The results revealed that the risk of DKD, low-eGFR, and albuminuria decreased with increasing quartiles across all models (*p* for trend <0.05). In model 3, which was fully adjusted, compared to the lowest quartile of OBS, the highest quartile was associated with a 39% reduction in the risk of DKD (OR = 0.61, 95% CI: 0.46-0.80), a 54% reduction in the risk of low-eGFR (OR = 0.46, 95% CI: 0.33-0.64), and a 32% reduction in the risk of albuminuria (OR = 0.68, 95% CI: 0.51-0.92).

**Table 2:** Weighted logistic regression analysis models showing the associations between OBS and DKD.

Variable	OBS levels quartile						*p* value	*p* for trend
	Q1	Q2	*p* value	Q3	*p* value	Q4		
	OR (95%CI)	OR (95%CI)		OR (95%CI)		OR (95%CI)		
OBS								
Unadjusted	1.00(ref)	0.81(0.65-1.01)	0.065	0.63(0.52-0.77)	<0.001	0.49(0.38-0.63)	<0.001	<0.001
Model 1	1.00(ref)	0.82(0.65-1.05)	0.112	0.68(0.55-0.85)	<0.001	0.58(0.44-0.76)	<0.001	<0.001
Model 2	1.00(ref)	0.80(0.62-1.04)	0.09	0.70(0.55-0.88)	0.003	0.59(0.45-0.78)	<0.001	<0.001
Model 3	1.00(ref)	0.81(0.63-1.05)	0.116	0.71(0.56-0.91)	0.006	0.61(0.46-0.80)	<0.001	<0.001
Dietary OBS								
Unadjusted	1.00(ref)	0.72(0.58-0.89)	0.003	0.65(0.53-0.81)	<0.001	0.49(0.39-0.62)	<0.001	<0.001
Model 1	1.00(ref)	0.73(0.58-0.92)	0.007	0.71(0.56-0.90)	0.004	0.60(0.46-0.77)	<0.001	<0.001
Model 2	1.00(ref)	0.71(0.56-0.90)	0.005	0.72(0.56-0.92)	0.008	0.60(0.46-0.77)	<0.001	<0.001
Model 3	1.00(ref)	0.73(0.57-0.92)	0.009	0.74(0.57-0.95)	0.017	0.61(0.47-0.79)	<0.001	<0.001
Lifestyle OBS								
Unadjusted	1.00(ref)	1.03(0.82-1.30)	0.77	0.75(0.62-0.92)	0.006	0.69(0.49-0.97)	0.033	<0.001
Model 1	1.00(ref)	0.95(0.74-1.22)	0.687	0.68(0.55-0.85)	<0.001	0.57(0.38-0.85)	0.006	<0.001
Model 2	1.00(ref)	0.95(0.74-1.23)	0.71	0.74(0.60-0.93)	0.009	0.68(0.44-1.05)	0.081	0.004
Model 3	1.00(ref)	0.96(0.75-1.23)	0.744	0.79(0.64-0.99)	0.04	0.74(0.47-1.15)	0.174	0.027

Model 1: Adjusted for age, sex, race, marriage status, education and family PIR.

Model 2: Adjusted for model 1 + hypertension, hyperlipidemia, cardiovascular disease, metabolic syndrome, insulin use.

Model 3: Adjusted for model 2 + AST, ALT, TC, TG, glycohemoglobin, fasting glucose, ALB.

OBS, oxidative balance score; DKD, diabetic kidney disease. Q, quartiles; OR, odds ratio; 95% CI, 95% confidence interval.

**Table 3:** Weighted logistic regression analysis models showing the associations between OBS and low-eGFR.

Variable	OBS levels quartile						*p* value	*p* for trend
	Q1	Q2	*p* value	Q3	*p* value	Q4		
	OR (95%CI)	OR (95%CI)		OR (95%CI)		OR (95%CI)		
OBS								
Unadjusted	1.00(ref)	0.99(0.77-1.28)	0.959	0.65(0.51-0.82)	<0.001	0.43(0.34-0.56)	<0.001	<0.001
Model 1	1.00(ref)	0.94(0.72-1.24)	0.67	0.60(0.45-0.79)	<0.001	0.43(0.31-0.59)	<0.001	<0.001
Model 2	1.00(ref)	0.94(0.70-1.25)	0.664	0.62(0.45-0.85)	0.003	0.44(0.32-0.62)	<0.001	<0.001
Model 3	1.00(ref)	0.97(0.72-1.31)	0.831	0.64(0.47-0.86)	0.004	0.46(0.33-0.64)	<0.001	<0.001
Dietary OBS								
Unadjusted	1.00(ref)	0.91(0.70-1.18)	0.48	0.69(0.54-0.89)	0.005	0.44(0.33-0.57)	<0.001	<0.001
Model 1	1.00(ref)	0.88(0.65-1.18)	0.378	0.68(0.50-0.92)	0.014	0.48(0.35-0.66)	<0.001	<0.001
Model 2	1.00(ref)	0.87(0.64-1.17)	0.347	0.68(0.49-0.94)	0.021	0.48(0.34-0.67)	<0.001	<0.001
Model 3	1.00(ref)	0.91(0.67-1.24)	0.549	0.69(0.49-0.96)	0.027	0.49(0.35-0.68)	<0.001	<0.001
Life OBS								
Unadjusted	1.00(ref)	1.36(1.04-1.77)	0.024	1.03(0.78-1.35)	0.85	0.98(0.68-1.40)	0.901	0.63
Model 1	1.00(ref)	1.03(0.78-1.37)	0.821	0.73(0.55-0.97)	0.031	0.56(0.37-0.87)	0.01	0.001
Model 2	1.00(ref)	1.04(0.79-1.38)	0.763	0.84(0.63-1.14)	0.262	0.76(0.48-1.19)	0.225	0.114
Model 3	1.00(ref)	1.02(0.77-1.36)	0.885	0.91(0.68-1.23)	0.536	0.85(0.53-1.37)	0.508	0.386

Model 1: Adjusted for age, sex, race, marriage status, education and family PIR.

Model 2: Adjusted for model 1 + hypertension, hyperlipidemia, cardiovascular disease, metabolic syndrome, insulin use.

Model 3: Adjusted for model 2 + AST, ALT, TC, TG, glycohemoglobin, fasting glucose, ALB.

OBS, oxidative balance score; DKD, diabetic kidney disease. Q, quartiles; OR, odds ratio; 95% CI, 95% confidence interval.

**Table 4:** Weighted logistic regression analysis models showing the associations between OBS and albuminuria.

Variable	OBS levels quartile						*p* value	*p* for trend
	Q1	Q2	*p* value	Q3	*p* value	Q4		
	OR (95%CI)	OR (95%CI)		OR (95%CI)		OR (95%CI)		
OBS								
Unadjusted	1.00(ref)	0.65(0.51-0.82)	<0.001	0.59(0.47-0.74)	<0.001	0.52(0.40-0.68)	<0.001	<0.001
Model 1	1.00(ref)	0.69(0.54-0.88)	0.003	0.67(0.53-0.85)	0.001	0.65(0.49-0.87)	0.004	0.004
Model 2	1.00(ref)	0.66(0.51-0.85)	0.002	0.69(0.54-0.88)	0.003	0.66(0.49-0.89)	0.006	0.01
Model 3	1.00(ref)	0.66(0.51-0.86)	0.002	0.70(0.55-0.90)	0.005	0.68(0.51-0.92)	0.012	0.021
Dietary OBS								
Unadjusted	1.00(ref)	0.58(0.45-0.74)	<0.001	0.60(0.48-0.77)	<0.001	0.53(0.41-0.69)	<0.001	<0.001
Model 1	1.00(ref)	0.62(0.48-0.80)	<0.001	0.68(0.53-0.87)	0.003	0.67(0.52-0.87)	0.003	0.01
Model 2	1.00(ref)	0.60(0.46-0.78)	<0.001	0.69(0.53-0.89)	0.005	0.67(0.51-0.88)	0.004	0.016
Model 3	1.00(ref)	0.61(0.47-0.78)	<0.001	0.71(0.55-0.93)	0.012	0.69(0.52-0.91)	0.009	0.032
Life OBS								
Unadjusted	1.00(ref)	0.86(0.68-1.09)	0.206	0.67(0.55-0.83)	<0.001	0.58(0.41-0.82)	0.002	<0.001
Model 1	1.00(ref)	0.85(0.66-1.09)	0.193	0.67(0.53-0.84)	<0.001	0.56(0.38-0.82)	0.003	<0.001
Model 2	1.00(ref)	0.84(0.65-1.09)	0.186	0.71(0.57-0.90)	0.004	0.65(0.43-0.97)	0.037	0.002
Model 3	1.00(ref)	0.86(0.67-1.10)	0.227	0.77(0.61-0.96)	0.022	0.69(0.46-1.05)	0.086	0.015

Model 1: Adjusted for age, sex, race, marriage status, education and family PIR.

Model 2: Adjusted for model 1 + hypertension, hyperlipidemia, cardiovascular disease, metabolic syndrome, insulin use.

Model 3: Adjusted for model 2 + AST, ALT, TC, TG, glycohemoglobin, fasting glucose, ALB.

OBS, oxidative balance score; DKD, diabetic kidney disease. Q, quartiles; OR, odds ratio; 95% CI, 95% confidence interval.

Moreover, weighted multivariate logistic regression was also used to investigate the impact of dietary OBS and lifestyle OBS on the risk of DKD, low-eGFR, and albuminuria. The results indicated that both dietary OBS and lifestyle OBS were considered protective factors, although there was no statistically significant difference between the highest and lowest quartiles of lifestyle OBS.

### 3.3 Analysis of restricted cubic spline regression

**Figure 2:**
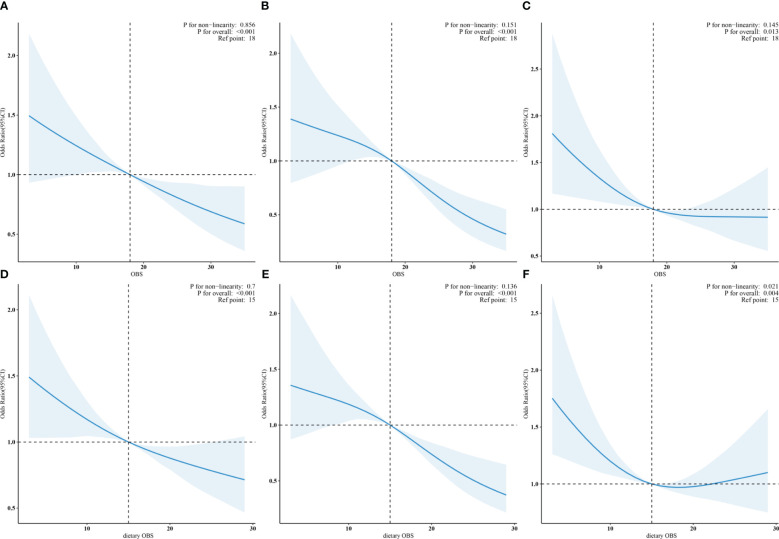
**(A–C)**: Linear associations of OBS with DKD, low-eGFR, and albuminuria. **(D, E)**: Linear trends in dietary OBS related to DKD and low-eGFR. **(F)**: Non-linear relation of dietary OBS to albuminuria.

Based on the results of the multivariate regression analysis, restricted cubic spline regression was employed to examine the relationship between OBS, dietary OBS, and the outcomes. After adjusting for all covariates, it was found that OBS was linearly associated with DKD, low-eGFR, and albuminuria. **Figures 2A–C** respectively display the trend of OR values for DKD, low-eGFR, and albuminuria decreasing with increasing OBS. Notably, although dietary OBS exhibited linear associations with DKD and low-eGFR (**Figures 2D, E**), it showed a non-linear association with albuminuria (*p* for non-linearity = 0.021, *p* for overall = 0.004). Specifically, **Figure 2F** illustrates that as dietary OBS increased, the risk of albuminuria initially decreased and then increased.

### 3.4 Analysis of subgroup

**Figure 3:**
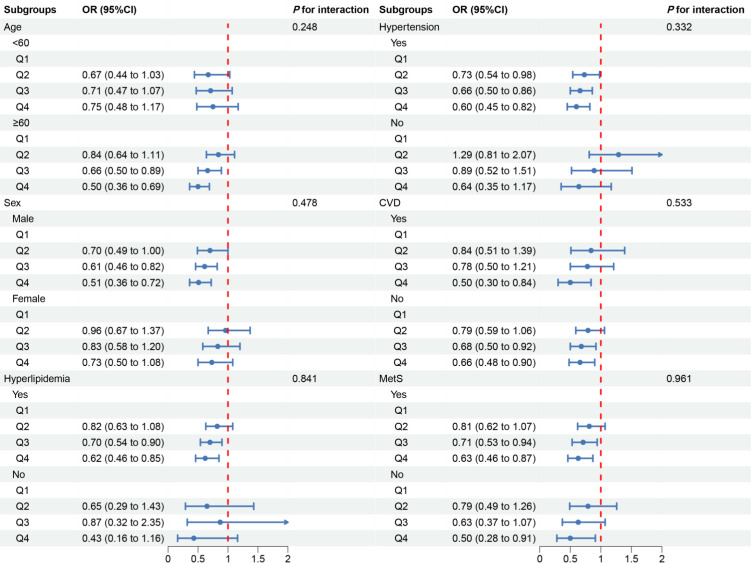
Associations between OBS and DKD in different subgroups. OR, odds ratio; 95% CI, 95% confidence interval; CVD, cardiovascular disease; Mets, metabolic syndrome. Except for the stratification component itself, each stratification factor was adjusted for all covariates.

**Figure 4:**
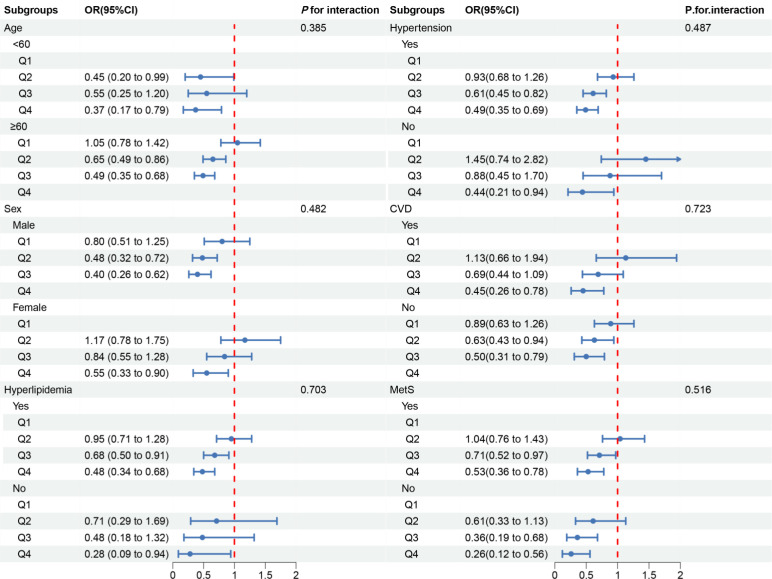
Associations between OBS and low-eGFR in different subgroups. OR, odds ratio; 95% CI, 95% confidence interval; CVD, cardiovascular disease; Mets, metabolic syndrome. Except for the stratification component itself, each stratification factor was adjusted for all covariates.

**Figure 5:**
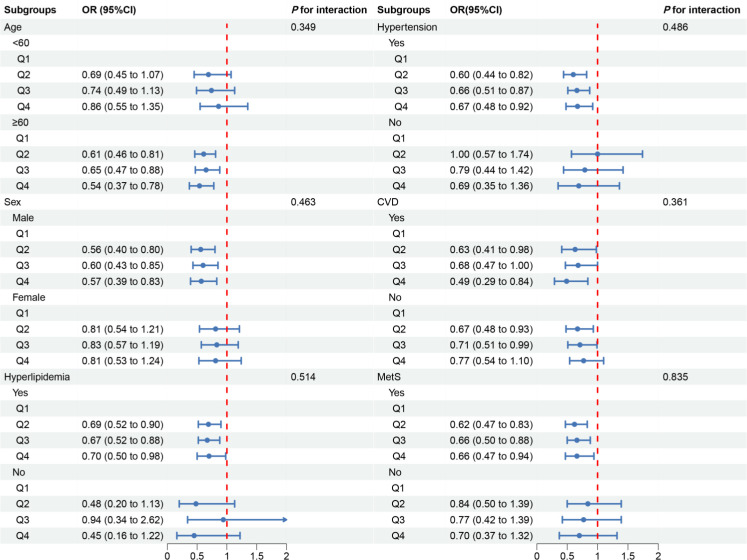
Associations between OBS and albuminuria in different subgroups. OR, odds ratio; 95% CI, 95% confidence interval; CVD, cardiovascular disease; Mets, metabolic syndrome. Except for the stratification component itself, each stratification factor was adjusted for all covariates.

In the subgroup analysis, we observed inconsistent associations. In the CVD and Mets subgroups, OBS was negatively correlated with DKD. However, in other subgroups, the correlation between OBS and DKD was only observed in individuals aged ≥60 years, males, those with hyperlipidemia or hypertension (**Figure 3**). A negative correlation between OBS and low-eGFR was present in all subgroups (**Figure 4**). The correlation between OBS and albuminuria was confined to subgroups of individuals aged ≥60 years, males, and those with hyperlipidemia, hypertension, CVD, or Mets (**Figure 5**). No interaction was found among the subgroups.

## 4 Discussion

We conducted a cross-sectional analysis of 5,389 participants from the NHANES, representing an estimated 23.6 million noninstitutionalized residents of the United States. To the best of our knowledge, this is the first study to explore the relationship between OBS and DKD. The results showed that OBS, as well as dietary OBS, was negatively associated with the risk of DKD, low-eGFR, and albuminuria, but no significant correlation was found between lifestyle OBS and these clinical outcomes. In the subgroup analysis, OBS was associated with DKD in both the CVD and Mets subgroups, in other subgroups, the association was observed only in individuals aged ≥60 years, males, those with hyperlipidemia or hypertension. Collectively, our study provides preliminary evidence for the relationship between OBS and DKD and offers new insights for future clinical and basic research.

In this study, we investigated the relationship between 20 oxidative stressors and DKD. Most of these oxidative stressors have been shown to prevent the development of DKD by modulating redox homeostasis. A cross-sectional study based on the MHANES database similarly found that maintaining an adequate antioxidant diet, as reflected in higher composite dietary antioxidant index (CDAI) levels, may lower the risk of DKD and mortality in diabetic individuals (29). This further supports our findings, yet differently, the OBS incorporates a more comprehensive range of antioxidant dietary factors while also considering lifestyle factors, which may provide a broader perspective for the comprehensive management of diabetic patients. Another study assessing the correlation between OBS, CDAI, and diabetic retinopathy (DR) found a negative association between OBS and DR after adjusting for potential confounders, but no such association was found in CDAI (30). It is well-known that both DR and DKD are microvascular complications of diabetes, with a shared pathogenesis involving endothelial dysfunction, and oxidative stress being one of the key pathological pathways (31). Additionally, some studies have focused on the impact of single factors on DKD, with varying results. In terms of dietary factors, an animal experiment (32) have suggested that crocin (water-soluble carotenoids) may reduce proteinuria levels by its antioxidant properties, enhancing the host’s antioxidant defense system, inhibiting inflammation and fibrosis. Ozcelik et al. (33, 34) found that zinc sulfate supplementation in diabetic rats can reduce kidney damage by activating metallothionein, which interact with Zn and iron to reduce ROS. A study (35) has demonstrated that vitamin C and E supplementation, as well as a combination of magnesium, zinc, and vitamins C and E, can decrease urinary albumin excretion levels and improve glomerular function in type 2 diabetes patients. Selenium supplementation for 12 weeks in patients with DKD has been shown to be beneficial for plasma glutathione peroxidase (GPx) and serum insulin levels (36). Folic acid, betaine, vitamins B6 and B12 have also been reported to delay the development of T2 DM by methylating degraded homocysteine (37). However, a meta-analysis suggests that neither vitamin B alone nor in combination improved renal function or blood pressure in diabetic patients. Due to the limited number and lower quality of included studies, these findings require further confirmation (38). In terms of lifestyle, exercise training has been shown to alleviate oxidative stress and inflammation in type 2 diabetic rats (39). Moreover, a lack of physical activity is more likely to cause obesity, exacerbating kidney damage. Smoking can lead to insulin resistance, induce advanced glycation end products, and increase kidney vascular permeability. Excessive alcohol consumption leads to the production of large amounts of ROS and reduces the antioxidant activity of GPx (40). Most of the above studies focusing on the association of a single factor with DKD are consistent with our results considering multiple factors together, suggesting that OBS may improve DKD by modulating oxidative homeostasis.

In the present study, restricted cubic spline regression showed that the OR of albuminuria showed a trend of decreasing followed by a slight increase with increasing dietary OBS, but overall the risk of albuminuria was still significantly lower in the higher dietary OBS group compared with the lower dietary OBS group, which may be related to the threshold effect of some antioxidants and interactions between antioxidants (41), beyond which antioxidants may exhibit pro-oxidant or depleting antioxidant properties. For example, the antioxidant properties of carotenoids depend on their interactions with vitamins E and C (42), and carotenoids may lose their antioxidant properties at high concentrations or high partial pressures of oxygen. Copper may also be pro-oxidant in certain environments, and vitamin E may play an inhibitory role in copper-dependent low density lipoprotein oxidation. In addition, lifestyle factors such as adequate physical activity, smoking cessation, alcohol restriction and weight control have beneficial antioxidant effects in a variety of diseases, including DKD. However, no direct relationship between lifestyle OBS and DKD, low-eGFR and albuminuria was found in the present study, which may be due to the insufficient degree of lifestyle variability in the study population or the possibility that lifestyle factors may influence DKD by interacting with other variables. Studies have reported clear benefits of an antioxidant lifestyle in reducing blood pressure and improving insulin resistance (43, 44). A cohort study conducted in China (45) explored the relationship between the triglyceride-glucose (TyG) index, a biomarker associated with insulin resistance, and the risk of CKD in hypertensive patients with abnormal glucose metabolism. The study found that a higher TyG index was associated with an increased risk of CKD, suggesting that an antioxidant lifestyle may indirectly influence the progression of DKD by affecting metabolic parameters such as the TyG index (46–48). For this reason, the present study also prefers to use the overall OBS to systematically assess the redox status of the organism and to avoid considering the effect of a single factor while ignoring the complex correlations and interactions between antioxidants.

In subgroup analyses, OBS was associated with DKD in both the CVD and Mets subgroups, in other subgroups, the association was present only in individuals aged 60 years or older, males and those with hyperlipidemia or hypertension. A cross-sectional study suggests that OBS is inversely associated with accelerated phenotypic ageing (49). Oxidative damage is considered to contribute to the progression of ageing and fundamental components of pathological pathways, which are thought to drive various age-related diseases (50), such as DKD. Hypertension, hyperlipidemia, and type 2 diabetes are common comorbidities. Compared to non-diabetic individuals, diabetic patients have twice the incidence of hypertension (51), and the specific pathogenesis is related to oxidative stress. Hyperglycemia, hypertension, and hyperlipidemia can all lead to increased vascular ROS generation, and oxidative stress activation promotes post-translational oxidation of proteins, mitochondrial dysfunction, etc., thereby causing cellular damage and vascular dysfunction (52), and leading to kidney injury. Furthermore, it has been reported that gender differences may also be one of the key factors affecting the progression of DKD (53). In animal models, estrogens can counteract renal fibrosis and apoptosis (54), while testosterone promotes inflammatory, apoptotic, and fibrotic processes (55), which may explain the significant association between OBS and DKD in male populations. However, these findings differ from human-based studies, which indicate that oral contraceptives and estrogen replacement therapy are associated with an increased risk of microalbuminuria and declining renal function (56, 57). Therefore, the different mechanisms of gender differences in DKD warrant further exploration. Furthermore, considering the possibility of multiple comparisons and type I errors, the results of subgroup analyses still need to be validated in future studies.

This study has several strengths. Firstly, it is based on the NHANES database, which employs a multistage complex probability sampling design and weighted adjustments, enhancing the representativeness and reliability of the study results—a crucial factor for improving the generalizability of our findings within the American context. Secondly, our study provides preliminary evidence of the correlation between OBS and DKD, with information on OBS being obtained solely through questionnaires and incorporating the comprehensive impact of 20 oxidative stress factors, which enables OBS to have the potential as a tool for diabetes risk stratification. This integration offers new insights for the development of personalized and more effective preventive strategies. Furthermore, our research paves the way for future longitudinal study designs and the exploration of the mechanisms by which OBS affects DKD. Confirmation and expansion of these findings will assist in the formulation or adjustment of public health policies, such as recommending specific antioxidant dietary and lifestyle practices for diabetes patients, which will contribute to the broader goal of reducing the global burden of DKD.

This study, despite employing weighted multivariate logistic regression models for adjustment and validating the stability of results through subgroup analyses, still has certain limitations. Firstly, the inherent nature of cross-sectional study restricts the establishment of a causal relationship between OBS and DKD. Acknowledging this limitation, we recommend that future research on the relationship between OBS and DKD should employ a longitudinal design. This would enable researchers to determine whether OBS precedes the development of DKD, thereby providing more compelling evidence for a potential causal relationship. Secondly, despite the stratified and multistage sampling methods used in NHANES aiming to produce a nationally representative sample, there may still be some potential selection biases, such as non-response bias, self-selection bias, and three-stage sampling bias. These biases could result in the study findings differing from the true situation in the overall population. Although we used the weighted methods recommended by NHANES guidelines to analyze the data, which may have corrected this bias to some extent, caution should still be exercised when interpreting the results. Additionally, the OBS data in this study were collected through a 24-hour food recall questionnaire, a convenient method but potentially subject to recall bias and selection bias. In future studies, we will work towards identifying relevant objective biological markers to reduce this bias. Thirdly, the study sample was limited to the US population, hence the generalizability of the results to other racial backgrounds requires further validation in diverse racial populations.

## 5 Conclusion

Our study findings indicated that higher levels of OBS and dietary OBS were associated with a reduced risk of DKD, low-eGFR, and albuminuria. These results provided preliminary evidence for the importance of adhering to an antioxidant-rich diet and lifestyle among diabetic patients and highlighted the potential utility in formulating feasible dietary and lifestyle recommendations for them. Consequently, the integration of OBS into clinical practice may represent an innovative strategy to mitigate the burden of DKD in diabetic patients. However, further large-scale prospective studies are necessary to validate and expand upon our observations.

## Data availability statement

The datasets presented in this study can be found in online repositories. The names of the repository/repositories and accession number(s) can be found below: The data employed in this study can be accessed through the NHANES website: https://www.cdc.gov/nchs/nhanes/.

## Ethics statement

The studies involving humans were approved by National Center for Health Statistics. The studies were conducted in accordance with the local legislation and institutional requirements. The participants provided their written informed consent to participate in this study.

## Author contributions

CL: Data curation, Formal analysis, Methodology, Software, Validation, Writing – original draft, Writing – review & editing. JY: Conceptualization, Data curation, Formal analysis, Methodology, Writing – review & editing. HL: Conceptualization, Methodology, Visualization, Writing – review & editing. YD: Formal analysis, Software, Visualization, Writing – review & editing. PH: Conceptualization, Methodology, Validation, Visualization, Writing – review & editing. JZ: Validation, Writing – review & editing. MZ: Data curation, Supervision, Visualization, Writing – review & editing.

## Supplementary material

Supplementary Table 1 Components of the oxidative balance score. OBS, oxidative balance score; A, antioxidant; P, prooxidant; RE, retinol equivalent; ATE, alpha-tocopherol equivalent; MET, metabolic equivalent.

Supplementary Table 2 The collinearity assessment outcomes. Family PIR, family poverty income ratio; TC, total cholesterol; TG, triglycerides; ALT, alanine aminotransferase; AST, aspartate aminotransferase; ALB, albumin.
