# Supplementary material for: Association between oxidative balance score and diabetic kidney disease, low estimated glomerular filtration rate and albuminuria in type 2 diabetes mellitus patients: a cross-sectional study
Source: Front Endocrinol (Lausanne). 2024 Jul 31;15:1412823. doi: 10.3389/fendo.2024.1412823 (PMC11322072; doi:10.3389/fendo.2024.1412823)
Supplement: Supplementary Table 1 — Components of the oxidative balance score. OBS, oxidative balance score; A, antioxidant; P, prooxidant; RE, retinol equivalent; ATE, alpha-tocopherol equivalent; MET, metabolic equivalent. [file Table_1.docx]

Supplementary Table 1. Components of the oxidative balance score.

| OBS components | Property | Male | | | Female | | |
| --- | --- | --- | --- | --- | --- | --- | --- |
|  |  | 0 | 1 | 2 | 0 | 1 | 2 |
| Dietary OBS components | | | | | | | |
| Dietary fiber (g/d) | A | ≤12.7 | 12.7-19.85 | ＞19.85 | ≤10.8 | 10.8-16.2 | ＞16.2 |
| Carotene (RE/d) | A | ≤54.542 | 54.542-180.083 | ＞180.083 | ≤55.306 | 55.306-187.736 | ＞187.736 |
| Riboflavin (mg/d) | A | ≤1.648 | 1.648-2.365 | ＞2.365 | ≤1.297 | 1.297-1.855 | ＞1.855 |
| Niacin (mg/d) | A | ≤19.989 | 19.989-28.585 | ＞28.585 | ≤15.032 | 15.032-21.265 | ＞21.265 |
| Vitamin B_6_ (mg/d) | A | ≤1.535 | 1.535-2.283 | ＞2.283 | ≤1.214 | 1.214-1.75 | ＞1.75 |
| Total folate (mcg/d) | A | ≤295.5 | 295.5-448 | ＞448 | ≤242.5 | 242.5-356 | ＞356 |
| Vitamin B_12_ (mcg/d) | A | ≤3.28 | 3.28-5.66 | ＞5.66 | ≤2.495 | 2.495-4.292 | ＞4.292 |
| Vitamin C (mg/d) | A | ≤40 | 40-91.65 | ＞91.65 | ≤38.833 | 38.833-87.3 | ＞87.3 |
| Vitamin E (ATE) (mg/d) | A | ≤5.405 | 5.405-8.69 | ＞8.69 | ≤4.435 | 4.435-7.067 | ＞7.067 |
| Calcium (mg/d) | A | ≤648 | 648-1006 | ＞1006 | ≤560.5 | 560.5-848.167 | ＞848.167 |
| Magnesium (mg/d) | A | ≤235.5 | 235.5-330.5 | ＞330.5 | ≤192.5 | 192.5-268 | ＞268 |
| Zinc (mg/d) | A | ≤8.96 | 8.96-13.255 | ＞13.255 | ≤6.645 | 6.645-9.777 | ＞9.777 |
| Copper (mg/d) | A | ≤0.971 | 0.971-1.38 | ＞1.38 | ≤0.812 | 0.812-1.121 | ＞1.121 |
| Selenium (mcg/d) | A | ≤92.35 | 92.35-130.85 | ＞130.85 | ≤70.1 | 70.1-99.05 | ＞99.05 |
| Total fat (g/d) | P | ＞93.42 | 62.45-93.42 | ≤62.45 | ＞70.515 | 46.348-70.515 | ≤46.348 |
| Iron (mg/d) | P | ＞17.3 | 11.82-17.3 | ≤11.82 | ＞13.638 | 9.392-13.638 | ≤9.392 |
| Lifestyle OBS components | | | | |  |  |  |
| Physical activity (MET-minute/week) | A | ≤720 | 720-2880 | ＞2880 | ≤504 | 504-1680 | ＞1680 |
| Alcohol (g/d) | P | ≥30 | 0-30 | None | ≥15 | 0-15 | None |
| Body mass index (kg/m^2^) | P | ＞33.2 | 28.1-33.2 | ≤28.1 | ＞35.63 | 29.5-35.63 | ≤29.5 |
| Cotinine (ng/mL) | P | ＞0.245 | 0.02-0.245 | ≤0.02 | ＞0.084 | 0.015-0.084 | ≤0.015 |

OBS: oxidative balance score; A: antioxidant; P: prooxidant; RE: retinol equivalent; ATE: alpha-tocopherol equivalent; MET: metabolic equivalent.
