# Supplementary material for: Association between oxidative balance score and diabetic kidney disease, low estimated glomerular filtration rate and albuminuria in type 2 diabetes mellitus patients: a cross-sectional study
Source: Front Endocrinol (Lausanne). 2024 Jul 31;15:1412823. doi: 10.3389/fendo.2024.1412823 (PMC11322072; doi:10.3389/fendo.2024.1412823)
Supplement: Supplementary Table 2 — The collinearity assessment outcomes. Family PIR, family poverty income ratio; TC, total cholesterol; TG, triglycerides; ALT, alanine aminotransferase; AST, aspartate aminotransferase; ALB, albumin. [file Table_2.docx]

| variables | GVIF | df | GVIF^(1/(2*df)) |
| --- | --- | --- | --- |
| Age | 1.326 | 1 | 1.151 |
| Sex | 1.226 | 1 | 1.107 |
| Race and ethnicity | 1.386 | 3 | 1.056 |
| Educational level | 1.323 | 2 | 1.073 |
| Marriage status | 1.238 | 2 | 1.055 |
| Family PIR | 1.287 | 2 | 1.065 |
| Hyperlipidemia | 1.112 | 1 | 1.054 |
| Hypertension | 1.122 | 1 | 1.059 |
| Cardiovascular disease | 1.118 | 1 | 1.057 |
| Metabolic syndrome | 1.211 | 1 | 1.1 |
| Insulin use | 1.161 | 1 | 1.077 |
| Glycohemoglobin, % | 2.398 | 1 | 1.548 |
| Fasting glucose, mmol/L | 2.229 | 1 | 1.493 |
| AST, U/L | 2.52 | 1 | 1.587 |
| ALT, U/L | 2.608 | 1 | 1.615 |
| TC, mmol/L | 1.359 | 1 | 1.166 |
| TG, mmol/L | 1.381 | 1 | 1.175 |
| ALB, g/dL | 1.137 | 1 | 1.066 |

Supplementary Table 2. The collinearity assessment outcomes
